# Human Gait Control Using Functional Electrical Stimulation Based on Controlling the Shank Dynamics

**DOI:** 10.32598/bcn.11.1.173.2

**Published:** 2020-01-01

**Authors:** Zohre Rezaee, Hamid Reza Kobravi

**Affiliations:** 1. Research Center of Biomedical Engineering, Mashhad Branch, Islamic Azad University, Mashhad, Iran.

**Keywords:** Gait, Rehabilitation, Spinal cord injuries, Muscle contraction Introduction

## Abstract

**Introduction:**

Efficient gait control using Functional Electrical Stimulation (FES) is an open research problem. In this research, a new intermittent controller has been designed to control the human shank movement dynamics during gait.

**Methods:**

In this approach, first, the three-dimensional phase space was constructed using the human shank movement data recorded from the healthy subjects. Then, three iterated sine-circle maps were extracted in the mentioned phase space. The three identified one-dimensional maps contained the essential information about the shank movement dynamics during a gait cycle. Next, an intermittent fuzzy controller was designed to control the shank angle. According to the adopted intermittent control strategy, the fuzzy controller is activated whenever the shank angle is far enough from the specific. The specific points are described using the identified iterated maps in the constructed phase space. In this manner, the designed controller is activated during a short-time fraction of the gait cycle time.

**Results:**

The designed intermittent controller was evaluated through some simulation studies on a two-joint musculoskeletal model. The obtained results suggested that the pattern of the obtained hip and knee joint trajectories, the outputs of the musculoskeletal model, were acceptably similar to the joints’ trajectories pattern of healthy subjects.

**Conclusion:**

The intriguing similarity was observed between the dynamics of the recorded human data and those of the controlled musculoskeletal model. It supports the acceptable performance of the proposed control strategy.

## Highlights

- The gait process was controlled with acceptable quality, while no desired trajectories were envisioned for joint movements.
- The intriguing similarity was observed between the dynamics of the recorded human data and the dynamics of the controlled musculoskeletal model.
- The proposed control strategy could imitate the gait-related motor control process.
- Adopting the proposed control strategy did not increase high frequency fluctuating joint trajectories despite its intermittent behavior.

## Plain Language Summary

The patients with spinal cord injury or stroke either cannot walk normally or cannot walk at all. So far, no clinical treatment approaches have been approved for gait recovery in such patients. Therefore, rehabilitation techniques are the most effective choices to help them. Functional Electrical Stimulation (FES) is an effective approach for movement recovery in paralyzed limbs based on the stimulation of neuromuscular systems. However, FES has limited performance owing to the time-varying and nonlinear behavior of muscles. So, using control algorithms is necessary to ameliorate the performance. In this study, a new control strategy, based on a new insight into controlling biological systems, has been proposed. Before the experimental study on the human, some preliminary evaluations should be done through the simulation studies. This study evaluated the effects of the proposed approach on a musculoskeletal model. The musculoskeletal model is used as a virtual patient. According to the study results, the proposed control strategy could control gait using FES with acceptable performance. Besides, the proposed control strategy can be used to implement the new version of the FES-based rehabilitation prosthesis for gait control in patients with spinal cord injury or some post-stroke conditions.

## 1. Introduction

Gait control is of critical importance in daily living activities. The stability of human gait is a complex process that can maintain an upright posture while walking against external perturbations. There are two basic needs for walking; first, the ability to stand and maintain balance, and second, the ability to take continuous and sequential steps. These two requirements are essential; however, other factors also affect walking (Lee & Guan, 2000). It is unclear which control mechanism is exactly adopted by the Central Nervous System (CNS) to maintain walking stability.

Nevertheless, gait rehabilitation indicates restoring the kinematic coordination pattern among the leg joint angles, the same as what is observed in healthy individuals. Functional Electrical Stimulation (FES) is a potentially effective strategy for gait restoration (Chen et al., 2013). Various controllers have been designed for gait correction in patients with CNS disorders using FES (Chen et al., 2013; O’Keeffe, Donnelly, Lyons, 2003; Lyons, Wilcox, Lyons, Hilton, 2000). For example, healthy activation patterns have been applied for drop foot correction in FES. However, this method requires the use of heavy large equipment and sensors (O’Keeffe et al., 2003; Lyons et al., 2000). In the mentioned study, the user’s step frequency was predicted using the designed system through 5 different methods. Then, the prediction method which had the best efficiency-accuracy was adopted in the FES system. In this study, the problem of energy efficiency was disregarded (Chen et al., 2013).

The optimal control of the ankle joint has been used for the Drop Foot Correction (DFC) in hemiplegic patients by FES (Benoussaad, Mombaur, Azevedo-Coste, 2013). To reduce energy consumption, no trajectory of the foot orientation was predefined or tracked (Benoussaad, Mombaur, Azevedo-Coste, 2013). A hybrid system consisting of a multi-channel FES stimulator and an Ankle-Foot Orthosis (AFO) was also proposed (Andrews et al., 1988). Supported walking control has also been explored by some researchers (Nekoukar & Erfanian, 2012). In one study, the adaptive fuzzy terminal sliding mode controllers were applied to control the joint angle trajectory and minimize upper body effort during the supported gait (Nekoukar & Erfanian, 2012). The controllers adjusted the lower muscle stimulation intensity in which the lower extremity walking pattern lies within a defined boundary of the reference trajectory (Nekoukar & Erfanian, 2012).

In another study, the joint angle trajectories were controlled in which moved toward the saddle cycle through the stable manifold in the phase space. However, a comprehensive model of human gait is required for identifying such a saddle cycle (Chunjiang, Suzuki, Kiyono, Morasso, & Nomura, 2014).

The emergence of desired kinematic coordination dynamics during the gait may be preferable, compared to the tracking the control of the joint angle trajectories during gait. The designed reference trajectories are usually periodic, while the gait is not a periodic process; it is a rhythmic process. Therefore, the present study aimed at controlling gait through a manner by which gait can be performed in compliance with a specific dynamic identified using the Poincare technique in lack of a comprehensive model of human gait. Besides, decreasing the stimulation energy has been regarded in this study.

**Figure 1.**
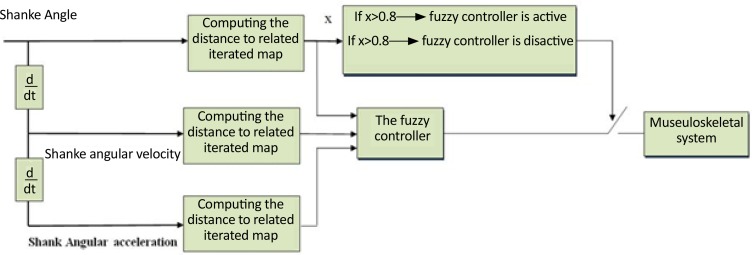
The block diagram describing the proposed intermittent control system

The dynamic control of some behaviors, such as quiet standing or goal tracking, has been conducted using the intermittent control strategy (Huryn, Croft, Koehle, & Loos, 2014; Loram, van de Kamp, Gollee, & Gawthrop, 2012; Inoue & Sakaguchi, 2013; Zenzeri, De Santis, & Morasso, 2014). Some researchers reported that compared to continuous controllers, intermittent controllers could control behaviors with lower stimulation energy (Huryn, et al., 2014). Therefore, the proposed controller designed in this study for human gait correction was intermittent. According to the designed strategy, a fuzzy feedback controller was activated whenever the system trajectories in the constructed state space were far enough from a specific region within the attractor of the state space. Figure 1 shows the block diagram of the applied proposed approach to design the control strategy.

## 2. Methods

In the proposed approach, the recorded data were analyzed in the phase space using an appropriate Poincaré section. It was expected that the gathered points describe the essential information. After gathering the points using the Poincare technique, a sine-circle map was identified that could describe the variations of these points relative to each other. Gait, as a biological process is not a periodic procedure; it is rather a rhythmic process. The sine-circle map was selected because it can present periodic, quasiperiodic, and chaotic behaviors. Accordingly, all speculated gait dynamics could be described by such mapping. According to the designed intermittent control strategy, the feedback controller was activated whenever the significant Euclidean distance existed between the shank angle value and the points described by an execrated sine-circle map in the state space. In this study, the distance threshold was selected to be 0.8. As a result, the controller forced the system to comply with specific dynamics identified within the attractor constructed by the healthy-subject data.

**Figure 2.**
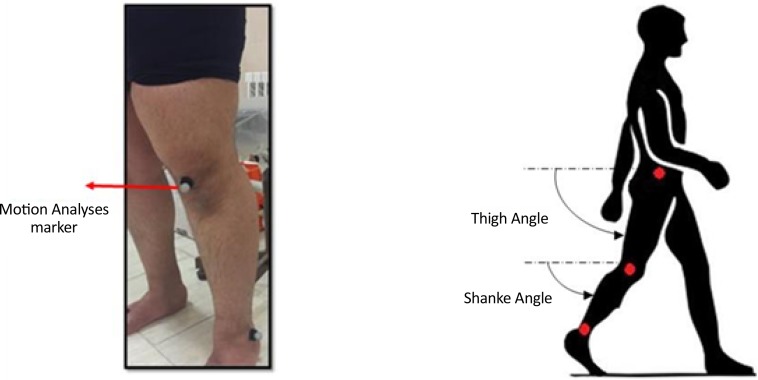
The placement of the markers for motion analysis

The human gait data recorded in this study were acquired from 5 healthy participants during 3 consecutive gait cycles. The study subjects were instructed to take steps in their normal gait speed and with their normal step length. The motion analyzer (Qualisys product) was used to record the desired angles. Moreover, the recording frequency was set at 100 Hz. Figure 2 shows the position of markers that were placed on the study participants’ body for tracking the shank and thigh movement during gait by the motion analyses system. Figure 2 also demonstrates the shank and thigh angles. Table 1 presents the demographic characteristics of the study subjects.

**Table 1.** The study subjects’ characteristics

**Subjects**	**Gender**	**Age (y)**	**Height (cm)**
1	Female	24	168
2	Female	24	165
3	Female	26	175
4	Female	29	168
5	Male	23	178

In this study, the angle, angular velocity, and angular acceleration accounted for the state variables. The state-space was constructed by plotting the state variables relative to each other. In other words, the shank angle, angular velocity, and angular acceleration were considered as the 3 state variables of the system. In such space, moving the system toward the desired dynamic could lead to the quantitative and qualitative control of gait; this is because the angle position and the angular velocity are controlled. The recorded data related to each gait cycle was analyzed separately. Then, after adopting Poincaré technique-based analyses in the phase space, the obtained results were compared.

**Figure 3.**
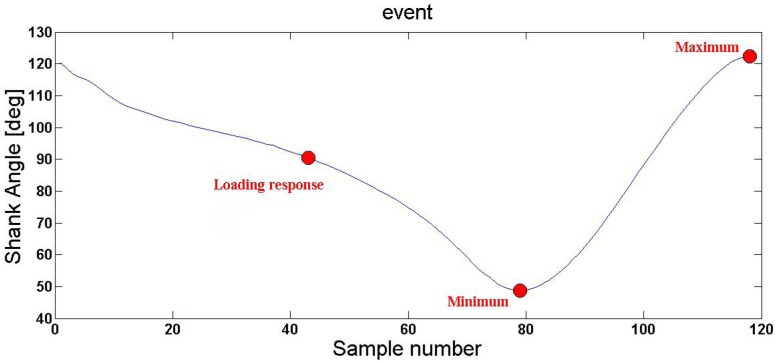
The demonstrated points show the timing of three gait events

Poincaré section is a technique applied for selecting the points within the system phase space containing the essential information concerning the system dynamics. Using methods like the Poincaré section helps with identifying the self-organizing biological system dynamics; accordingly, we could achieve a better understanding of the complexity of nonlinear dynamical systems. At this step, the goal was to find the surface equation based on the plotted attractor in each step. The surface equation was obtained using the calculated normal vector method. First, the values of angle, angular velocity, and angular acceleration of the shank must have been normalized. For this purpose, the values of angle, angular velocity, and angular articulation of the shank were mapped to the interval [−1, +1] by dividing the maximum value. Then, three points on the shank trajectory (Figure 3), corresponding to three gait events were selected. The three gait events were as follows:

Loading response point: This point is equivalent to the loading response when the whole body’s weight is distributed on the foot and foot position is vertical. At this moment, the shank angle is about 90 degrees. This phase is the end of the previous cycle and the start of the next cycle. If this phase is not performed correctly, the human balance may be jeopardized. Minimum point: At this point, the shank angle value is at minimal possible. It is when the knee is completely flexed.

Maximum point: At this point, the shank angle value is at maximal possible. It is when the knee is in full extension. The aforementioned selected points in the phase space were used to obtain the normal vector. As mentioned above, the surface can be generated using the normal vector, as follows: Selecting the three points (z_1_,y_1_, x_1_), (z_2_, y_2_, x_2_) and (z_3_, y_3_, x_3_) with the conditions listed above to select one of these points as the origin. Forming two vectors that pass through these points. Calculating the external product of two obtained vectors.

The external product outcome was a vector, i.e. vertical on the two other vectors. Accordingly, the normal vector was obtained. Then, the equation of the surface was calculated as the following equation:

1. $$a\left(x-x_{0}\right)+b\left(y-y_{0}\right)+c\left(z-z_{0}\right)=0$$

**Figure 4.**
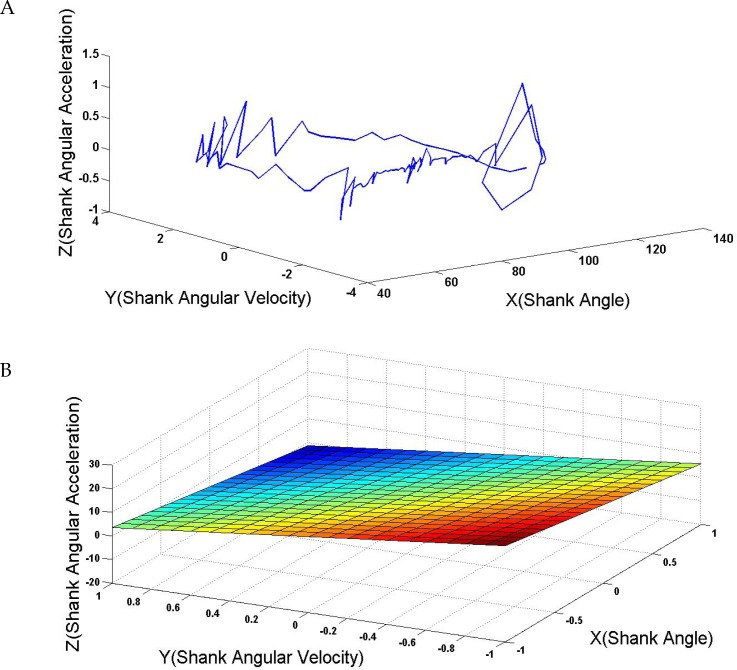
A. A state space constructed using recorded gait data; B. A designed Poincaré section

In Equation 1, (a, b, c) and (z_0_, y_0_, x_0_) represent the coordinates of the normal vector and the origin, respectively. Figure 4A shows a constructed sample state-space using the shank angle recorded during one gait cycle. Figure 4B shows a Poincaré surface identified using the human data recorded during a stride.

The Mean±SD values of the Poincaré surface parameters identified using the human data recorded during the different strides relating to different subjects were calculated and analyzed. Table 2 illustrates the calculated total Mean±SD values related to all recorded data. As per Table 2, the parameters of the identified Poincaré sections using the different data recorded during different gait cycles and relating to different subjects were close. Such result is intriguing, because the subjects walked normally, and no restrictions were considered in conducting the experiments.

**Table 2.** The computed coefficients of the Poincare plane equation

**Mean±SD**		

**A**	**B**	**C**
−0.18±0.09	−0.09±0.08	−0.10±0.02

As mentioned above, after constructing the state spaces, an appropriate Poincaré section was used to collect some points containing rich information concerned the gait dynamics. Then, a proper iterated map was identified to model the variations of the collected point relative to each other. A proper map described the complex emergent properties, including frequency locking, quasiperiodic, and chaotic behaviors. This is because gait is a cyclic but not necessarily a periodic process. Accordingly, the sine-circle map was selected, because it can describe the emergent mentioned above properties based on a simple theoretical and numerical framework (Jensen, Bak, & Bohr, 1984; Kelso, 1991; Nayak & Gupte, 2010).

The sine-circle is a nonlinear map functioning with the nonlinearity taking the specific form of a sine function:

2. $$\theta_{n+1}=\theta_{n}+\Omega -\frac{K}{2\pi }\sin\left(2\pi \theta_{n}\right)$$

In Equation 2 Ω and K determine the frequency ratio and the strength of the nonlinearity, respectively.

For numerical simplification, instead of identifying a proper three-dimensional map, three one-dimensional maps were identified in the phase space. The map parameters were estimated using the least square error method.

According to the proposed intermittent control, the feedback controller is activated when the value of the shank angle is significantly far from the points modeled by an identified sine-circle. Once the feedback controller is activated, the generated control signal causes to convergence the trajectories toward the identified sine-circle map in the phase space. Otherwise, the control signal will be equal to zero. This threshold’s value was selected as 0.8 using a trial and error approach. As a result, the controller was activated only for a short period during some phases of each gait. The control signal determines the muscle stimulation intensity; therefore, such a strategy can stave off the onset of muscle fatigue and reduces energy consumption.

**Figure 5.**
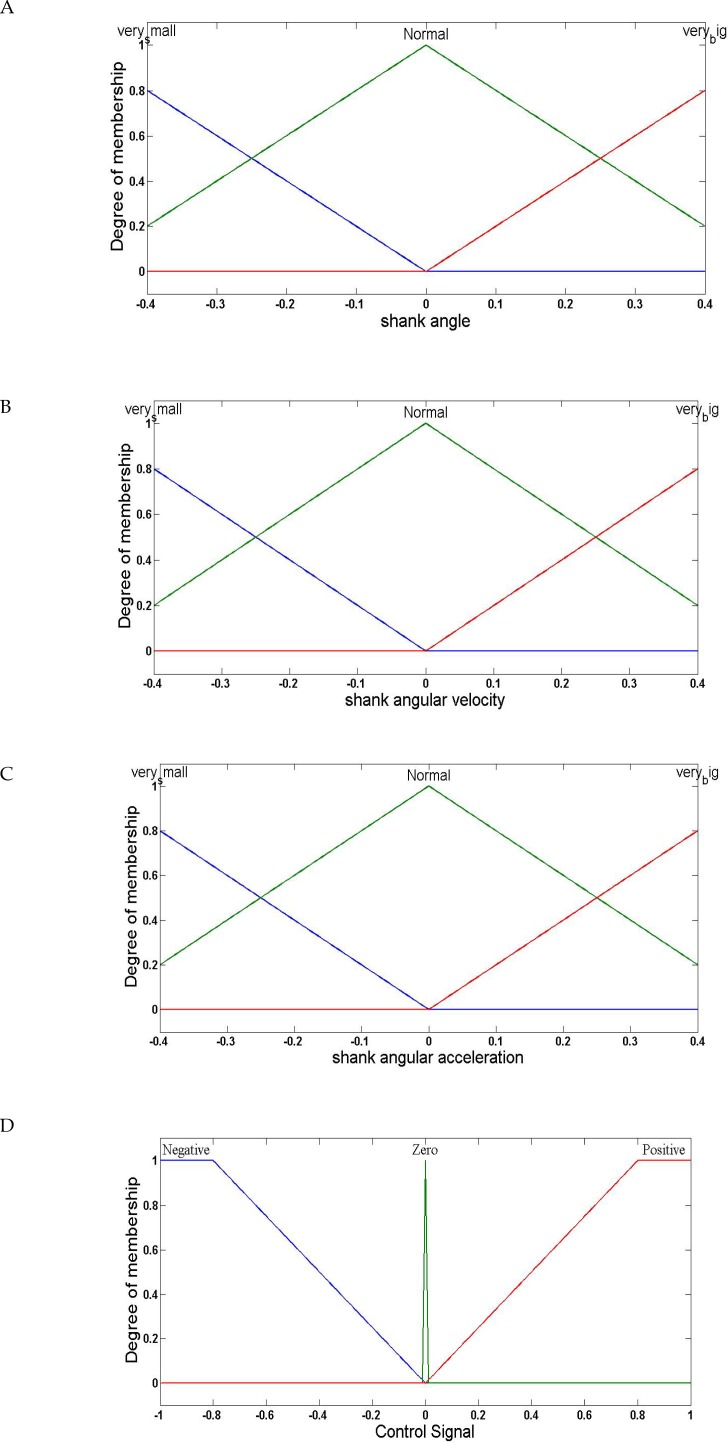
The assigned fuzzy membership functions A .To the shank angle as a fuzzy input variable; B. Shank angular velocity as a fuzzy input variable; C. Shank angular acceleration as a fuzzy input variable; and D. Control signal as the output variable of the fuzzy controller

The fuzzy controller was used as the feedback controller. The designed fuzzy controller had three inputs and one output. The spatial distance value between the system trajectories and identified sine-circle maps were three inputs of the controller. The controller output adjusted the stimulation intensity of the signal delivered to the 4 muscles involved in the gait. Figure 5 shows the assigned fuzzy membership functions.

The singleton fuzzier, product inference engine, and the center of gravity defuzzification method accounted for the fuzzy system. The specifications of the membership functions assigned to the controller output and inputs were selected using a trial and error approach. Three membership functions were assigned to each input and output variable. The extracted fuzzy rules were as follow: If the shank position is very small and shank velocity is very small, and shank acceleration is very small, then output is negative. If the shank position is normal and shank velocity is normal, and shank acceleration is normal, then output is zero. If the shank position is very big and shank velocity is very big, and shank acceleration is very big, then output is positive.

**Figure 6.**
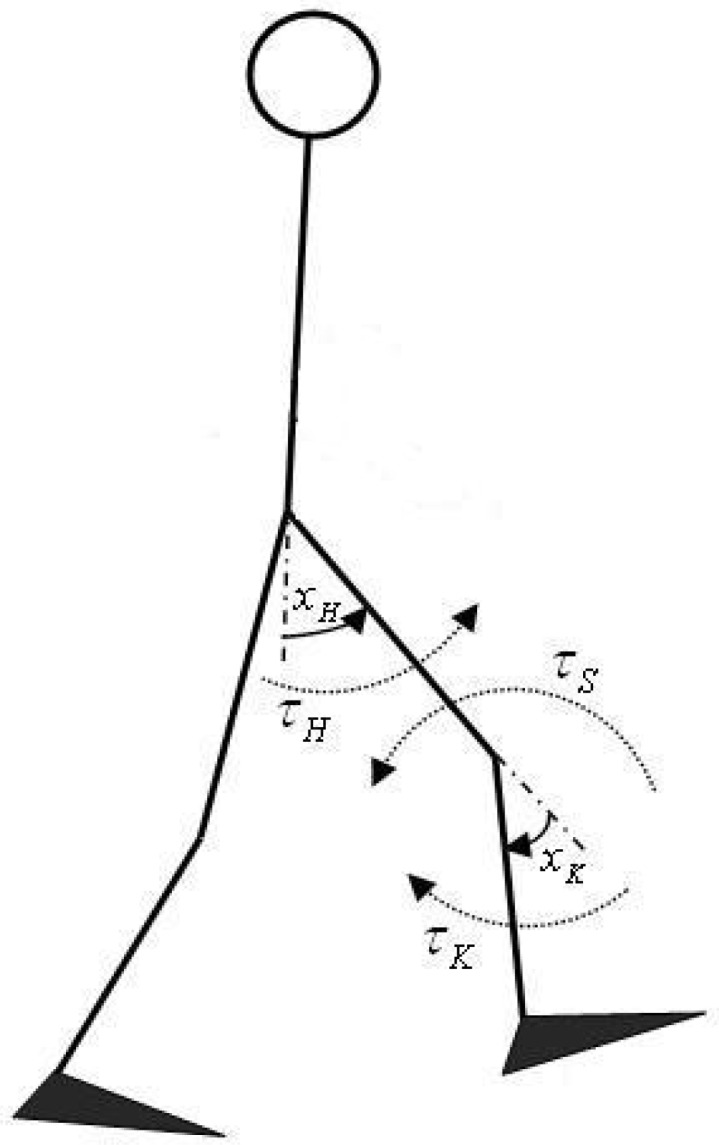
The skeletal parts of the musculoskeletal model used in simulation studies

A musculoskeletal model describing the bipedal locomotion was used for simulation investigations (Figure 6). In this model, the pairs of monoarticular muscles acting around the knee and hip joints account for the muscular part. The ankle and phalangeal joints were excluded from the model. More details about this model are described by prior research (Popović, Stein, Oğuztoreli, Lebiedowska, & Jonić, 1999; Nekoukar & Erfanian, 2011).

The following differential equations describe the dynamics in Equation 3 and Equation 4:

3 $$\begin{array}{l} M\;\;\ddot{x}(t)+C\dot{x}(t)+G+\tau_{fd}=\tau (t)+V(t)\\ \tau_{i}(t)=\tau_{i}^{f}(t)-\tau_{i}^{e}(t)-{\left(t\right)}_{i}^{r}i=k,H \end{array}$$

4 $$\begin{array}{l} \tau_{T}(t)=-\tau_{K}(t)\\ \tau_{S}(t)=\tau_{K}(t)+\tau_{H}(t) \end{array}$$

The equations for the active flexion (f) and extension (e) torques at the knee (K) and hip (H) joints have the following form (Equation 5):

5 $$\begin{array}{l} \tau_{j}^{i}(\text{t})=\left({\text{c}}_{i2}^{j}{\text{x}}_{i}^{2}+{\text{c}}_{i1}^{j}{\text{x}}_{\text{i}}+c_{i0}^{j}\right).{\text{g}}_{j}^{i}\left(\dot{x}\right).{\text{a}}_{j}^{i}\\ i=k,H\;\;\;j=f,e \end{array}$$

The X(t)=[x_K_ x_H_] and ẋ(t)=[ẋ_K_ ẋ_H_] are joint angles and angular velocities. The M and C are the inertia matrix and the generalized Coriolis matrix, respectively. G is a vector of gravity, T_fd_ fd is a vector of ground reaction torque, is white-noise modeling of the lumped time-varying system uncertainty and τ(t)=[τ_S_ τ _T_ ] is a vector of torques acting at the shank and thigh segments; τ^f^_i_(t) and τ^e^_i_(t) are the flexor and extensor torques, respectively. The τ^r^_i_ (t) is the resistive torque produced by passive tissues crossing the joints, and τ_i_(t) is the torque acting at the hip and knee joints. The signals are the normalized values of the activation of the involved muscles. The parameters c^j^_ik_j(k=0,1,2) determine the maximum torque generated in the isometric conditions (Popović et al., 1999; Nekoukar & Erfanian, 2011; Došen & Popović, 2009). The normalized torques against joint angular velocity are provided by previous research (Popović et al., 1999; Nekoukar & Erfanian, 2011) ( Equation 6):

6. $$\begin{array}{l} g_{i}^{f}\left({\dot{x}}_{i}\right)=\begin{array}{cc} \begin{array}{c} b_{i1}\\ 0\\ 1 \end{array} & \{\begin{array}{cc} {\dot{x}}_{i}<\frac{1-b_{i1}}{b_{i2}^{f}} & \\ \frac{1}{b_{i2}}\leq {\dot{x}}_{i} & \\ b_{i2}{\dot{x}}_{i} & \frac{1-b_{i1}}{b_{i2}}\leq {\dot{x}}_{i}<\frac{1}{b_{i2}} \end{array} \end{array}\\ g_{i}^{e}({\dot{x}}_{i})=\{\begin{array}{cc} b_{i4} & \frac{b_{i4-1}}{b_{i3}}\leq {\dot{x}}_{i}\\ 0 & {\dot{x}}_{i}\leq \frac{-1}{b_{i3}}\\ 1+b_{i3}{\dot{x}}_{i} & \frac{-1}{b_{i3}}\leq {\dot{x}}_{i}<\frac{b_{i4-1}}{b_{i3}} \end{array} \end{array}$$

Muscle activation dynamics can be modeled by the following Došen & Popović, 2009) ( Equation 7):

7. $$\left\{ \begin{array}{l} \left[u_{i}/\tau_{\mathrm{act}}+(1-u_{i})/\tau_{\mathrm{deact}}\right]\left(u_{i}-a_{i}\right)u_{i}\geq a_{i}\\ (u_{i}-a_{i})/\tau_{\mathrm{deact}}u_{i}<a_{i}\;\;\;\;\;\;\;\;\;\;\;\;\;\;\;i=1,2,3,4 \end{array}\right.$$

The parameters τ_act_ and τ_deact_ were time constants for activation and deactivation, respectively. The activation and deactivation time constants were 20 and 60 ms, respectively. The variables were the normalized values of intensity of the stimulation signal delivered to the muscles. The resistive torques, τ^r^_i_(t), were given by the following format (Popović et al., 1999; Nekoukar & Erfanian, 2011) (Equation 8):

8. $$\begin{array}{l} {\tau^{r}}_{K}(t)=d_{11}\left(x_{K}-x_{K0}\right)+d_{12}{\dot{x}}_{K}+d_{13}e^{d_{14}x_{K}}-d_{15}e^{d_{16}x_{K}}\\ {\tau^{r}}_{K}(t)=d_{21}\left(x_{H}-x_{H0}\right)+d_{22}{\dot{x}}_{H}+d_{23}e^{d_{24}x_{H}}-d_{25}e^{d_{26}x_{K}} \end{array}$$

Further details and the set of parameters (k=0,1,2)C^j^_ik_, (k=1,2,3,4) b_ik_ and (k=1,2,3,4,5,6)d_ik_ were obtained from a previous study (Popović et al., 1999; Došen & Popović, 2009).

## 3. Results

The current study analyzed the generalizability of the identified maps. The Mean±SD values of identified map parameters using the human data related to diffident gait cycles recorded from different subjects were computed. Table 3 presents the calculated total Mean±SD values related to all recorded data. The calculated SD of Ω values, i.e. related to the frequency ratio were small in comparison to the computed mean value; thus, the walking rhythm was similar in different subjects. Accordingly, the reference maps were designed using the mean value of the identified map parameters, as per Table 3.

**Table 3.** The parameters of three identified sine-circle maps

**Mean±SD**			

	**K**		Ω
K1	0.78±0.12	Ω1	0.61±0.05
K2	6.49e-008±1.40e-007	Ω2	0.02±0.03
K3	0.33±0.34	Ω3	0.03±0.02

Before interpreting the achieved results, it should be noted that in the designed control strategy, the control signal, as the muscle stimulation intensity, simultaneously stimulated 4 muscles.

Nevertheless, since each muscle group has different parameters, different excursion torques were generated through the joints. Such applied strategy is a simple agonist-antagonist muscle co-activation. In this manner, a favorable movement can be produced. Muscle co-activation is important in joint stabilization (Le, Best, Khan, Mendel, & Marras, 2017).

**Figure 7.**
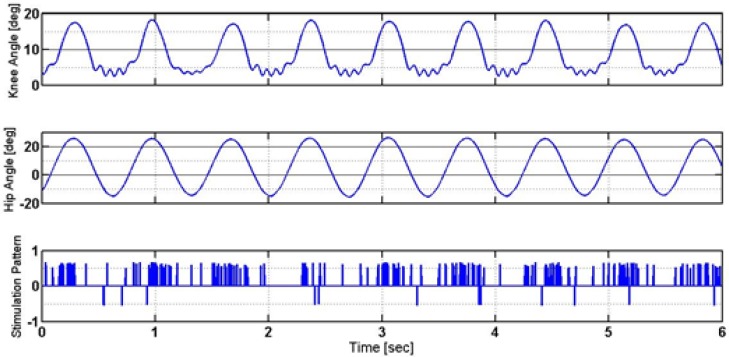
The trajectories of the knee and hip angles, and generated stimulation pattern

According to Figure 7, the control signal has an intermittent On-Off pattern. Besides, the hip joint and knee joint had a cyclic movement pattern. Eliciting such cyclic movement is intriguing concerning that no desired joint movement trajectories were envisioned. It is unlike what is usually performed in classical control. However, more quantitative and qualitative evaluations were required. Therefore, some different evaluations were conducted, i.e. elaborated in the following subsections.

The time duration of one stride in a healthy person during walking at a normal speed is about 1.2 s (Peggy & Bertoti, 2012). In this study, due to applying the designed intermittent controller, the stride time in the controlled musculoskeletal model was approximately 1.4 s. In other words, the designed intermittent controller could successfully control the walking process. This is because the observed stride time difference was about 0.2 s, compared to a healthy person; such time difference cannot significantly undermine the gait quality or jeopardize the subject’s stability. Additionally, the obtained knee and hip trajectories lie within their anatomical range (Jacquelin Perry, 1992). In other words, the control strategy automatically restricted the motion of the knee angle and hip angle to move within the normal range without the need to consider any control constraints.

The similarity between the dynamics of the recorded data and the dynamics of observed behavior related to the controlled musculoskeletal model could further clarify the ability of the proposed control strategy to imitate the gait-related motor control process. Two quantitative-qualitative features were extracted from the thigh and shank trajectories generated through the simulation studies. Then, these data were compared with the corresponding features extracted from the real data. Using these features, the elicited coordination dynamics between the thigh movement and shank movement was analyzed. Correlation Dimension (CD) and Lyapunov Exponent (LE) were computed and analyzed.

LE is a quantity characterizing the separation rate of nearby trajectories (Hilborn, 2000). Such a nonlinear feature can be considered as a feature containing some information concerning the system (signal) dynamics. Therefore, it was selected for dynamic comparison. Correlation dimension was chosen to focus on the geometric aspect of the joint angle trajectories. The correlation dimension has a computational advantage because it directly provides a computationally, simpler dimension for the trajectory points (Hilborn, 2000). The correlation dimension of the thigh and shank trajectories, like the one-dimensional trajectories, was calculated. The correlation dimension was calculated as follows (Hilborn, 2000):

9. $$C(R)=\{\begin{array}{c} D_{c}=\underset{R\to 0}{\lim}\frac{\log C(R)}{\log(R)}\\ \frac{1}{N}\sum_{i=1}^{N}p_{i}(R)\\ p_{i}(R)=N_{i}/N-1 \end{array}$$

In Equation 9, N indicates the number of trajectory points and C(R) is called the correlation sum, and the is defined as the relative number of points that lie within the distance of the point. The LE was also extracted from the time series of each time trajectory (Wolf, Swift, Swinney, & Vastano, 1985); i.e. thigh movement trajectory and shank movement trajectory, respectively.

Initially, the inter-subject and inter-trial variability of these features were analyzed. The Mean±SD values related to all recorded data were computed (Table 4).

**Table 4.** The Correlation Dimension (CD) and Lyapunov Exponent (LE)

**Mean±SD**			

**x**	**y**	**LEx**	**LEy**
1.85±0.01	1.78±0.02	2.10±0.12	2.64±0.17

Real data: Shank (x), Thigh (y)

The gait data collected from study subjects amounted to 15 gait cycles. Next, LE and correlation dimensions were extracted from the generated shank and thigh trajectories. The mentioned trajectories were generated through the simulation study on the musculoskeletal model (Table 5).

**Table 5.** The Correlation Dimension (CD) and Lyapunov Exponent (LE)

**x**	**y**	**LEx**	**LEy**
1.63	1.76	2.38	2.20

Real data: Shank (x), Thigh (y)

The computed difference between the mean value of the features extracted from the human data and those of the features extracted from the output of controlled musculoskeletal model were equal to 0.22 (Dcx), 0.02 (Dcy), 0.28 (LEx), and 0.44 (LEy). The computed differences were either comparable with the calculated standard deviations or were <50% of the mean value (Table 5). Therefore, the values of the correlation dimension and LEs extracted from the recorded data of a healthy person and the generated thigh and shank angle trajectories, as the output of the controlled musculoskeletal model, were also comparable. Such results highlight the similarity between the motion dynamics elicited due to controlling the musculoskeletal model, the adopted control strategy, and the motion dynamics that emerged from the kinematic synergy between two involved joints in healthy subjects during the normal walking were used.

**Figure 8.**
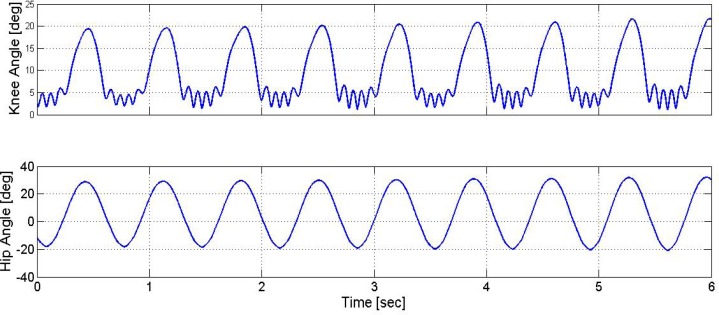
The trajectories of the knee and hip angles, and generated stimulation pattern The range of the assigned membership functions to the input and fuzzy output variables were increased as much as 0.2.

**Figure 9.**
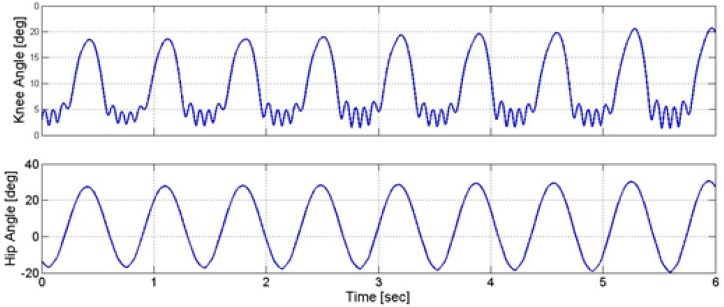
The trajectories of the knee and hip angles, and generated stimulation pattern The range of the assigned membership functions to the input and fuzzy output variables were increased as much as 0.4.

The parameters of the fuzzy membership functions were selected in a manner that the extracted fuzzy rule was complete, consistent, and continues. Next, the sensitivity analysis of the fuzzy controller parameters was carried out. For performing such an assessment, the range of the assigned membership functions was increased to 0.2 by 0.2 (in two steps). Besides, at each step, the controller performance was assessed again. Figure 8 and Figure 9 show the relevant results.

The computed differences were either comparable with the calculated standard deviations or were <50% of the mean value (Table 5). Therefore, the generated thigh and shank angle trajectories, as the output of the controlled musculoskeletal model, were also comparable. Such results can prove that significantly changing the parameters had no impact on controller performance. Thus, significant changes in the parameters had no impact on controller performance.

In the final stage, two features, including the correlation dimension and LEs, were extracted from the shank and thigh trajectories obtained through the simulation study related to controlling the musculoskeletal model. Table 6 and Table 7 present the features calculated in the context of changing the range of fuzzy membership functions.

**Table 6.** The Correlation Dimension (CD) and Lyapunov Exponent (LE)

**x**	**y**	**LEx**	**LEy**
1.73	1.74	1.78	2.12

Simulation data: Shank (x), Thigh (y)

The range of the assigned membership functions to the input and fuzzy output variables were increased as much as 0.2.

**Table 7.** The Correlation Dimension (CD) and Lyapunov Exponent (LE)

**x**	**y**	**LEx**	**LEy**
1.70	1.74	1.30	2.44

Simulation data: Shank (x), Thigh (y)

The range of the assigned membership functions to the input and fuzzy output variables were increased as much as 0.4.

## 4. Discussion

The intermittent control is a control paradigm suggested for biological control systems using discontinuous feedback (Craik, 1947; Vince, 1948; Neilson & Neilson, 2005; Bye & Neilson, 2008). Nevertheless, such a control paradigm has only been applied for postural balance control (Huryn, et al., 2014). In this study, an intermittent feedback controller has been developed to explain the gait control process. The control attitude was applied based on the restoration of kinematic coordination between the knee joint and hip joint. The movement dynamics of shank depends on knee joint position and hip joint position; thus, the control approach has determined the stimulation intensity of muscles to elicit a desired joint movement dynamic. The appearance of such desired dynamics indicates the emergence of the desired kinematic coordination between the involved joints during gait.

In contrast to the usual feedback control strategy, no desired trajectories were envisioned for joint movements. Instead, the controller tried to preserve the trajectories in the shank related state space within the specific region versus the location of the point that lies on the identified maps. While no designed desired trajectory was used, the closed-loop system remained stable, and the observed joint angle trajectories were rhythmic, the range of motion and cycle time of which were close to corresponding values extracted from human data. Such results suggest that controlling the shank movement dynamics could increase acceptable joint angle movement, even without using the desired trajectory. Accordingly, the control process could be feasible, in practice, using only one recording sensor measuring the shank position. Besides, the discontinuous control signal did not increase high frequency fluctuating joint trajectories. Such a result supports that if the intermittent controller could properly determine the activation timing of coupled muscles, discontinuous control signals do not elicit the fluctuation.

One important aspect of neuromuscular system performance is the antagonistic muscle coactivation process. This process is necessary for smoothing the joint movements. Some antagonistic muscle coactivation approaches have been proposed (Kobravi & Erfanian, 2009). However, in this study, a simple approach was applied based on interpreting the role of the antagonist muscle. The antagonist muscles could smooth the agonist muscle length variations during the movement. Accordingly, the stimulation signal was simultaneously delivered to 4 muscles. At each sample time, depending on the value of output error computed in the phase space of shank, the determined stimulation intensity caused a smooth flexion movement or smooth extension movement at joints. Smooth joint movement indicated the lack of abrupt joint movement. This phenomenon could be attributed to applied muscle coactivation strategy.

The comparison between the output of the controlled musculoskeletal model and subject-related data could reflect the similarity between the dynamics that arise from adopting the proposed control strategy and the coordination dynamics of involved joints during human gait. This finding was important, because the identified iterated maps, as the models describing the desired gait dynamics, were identified using human data. Therefore, quantitative and qualitative comparisons were carried out. The closeness values of the computed qualitative and quantitative measures (Table 4 and Table 5) indicated that the proposed controller, i.e. responsible for controlling the movement dynamics instead of controlling the output directly, in contrast, to control strategies from classical control approaches, has had acceptable performance. This could broaden our insights; because it can alter our conventional insight into the control of dynamic systems, like musculoskeletal systems; their dynamics emerged from interaction among the coupled sub-systems. Each muscle-joint could be considered as a sub-system.

Finally, by controlling the skeletal segment, the movement emerged from kinematic coordination among some joints; instead of controlling each joint separately may be an intriguing attitude. This is because, in this approach, not only the joint coordination dynamics are controlled, but also each joint movement is controlled indirectly. Furthermore, the intermittent control strategies could postpone the occurrence of muscle fatigue elicited from functional electrical stimulation and reduce energy consumption. Such an attitude could be regarded as a new frontier in controlling human gait using functional electrical stimulation. However, for practical implementations, it is required to simultaneously measure the position, angular velocity, and angular acceleration of the shank. It can be assigned as a practical limitation. However, the next step of this research path is preparing to provide an experimental setup for evaluating the proposed control strategy effects on patients.

## 5. Conclusion

The present research adopted a new control attitude based on intermittent strategy using the fuzzy logic system for gait control. The main idea behind this study lies in the fact that the desired movement indicates eliciting proper coordination among the involved joint. We have designed and evaluated an intermittent neural feedback controller to confine the joint angle trajectories within a specific region of state space, i.e. described by three iterated sine-circle maps. This process was conducted through the electrical stimulation of knee and hip muscles. The elicited movement dynamics of the controlled musculoskeletal model, as a virtual subject, was comparable with the human data. The future works could focus on using the adaptive controller and designing the soft switching mechanism for activating and deactivating the controller.
